# Fear of predation alters clone-specific performance in phloem-feeding prey

**DOI:** 10.1038/s41598-017-07723-6

**Published:** 2017-08-09

**Authors:** Mouhammad Shadi Khudr, Oksana Y. Buzhdygan, Jana S. Petermann, Susanne Wurst

**Affiliations:** 1 Faculty of Biology, Medicine and Health, The University of Manchester, Michael Smith Building, M13 9PT Manchester, UK; 2 Institute of Biology, Freie Universität Berlin, Königin-Luise-Straße 1-3, 14195 Berlin, Germany; 3 Berlin-Brandenburg Institute of Advanced Biodiversity Research (BBIB), D-14195 Berlin, Germany; 4 Department of Ecology and Evolution, University of Salzburg, Hellbrunnerstraße, A-5020 Salzburg, Austria

## Abstract

Fear of predation has been shown to affect prey fitness and behaviour, however, to date little is known about the underlying genetics of responses to predator-associated risk. In an effort to fill this gap we exposed four naïve clones of green peach aphid (*Myzus persicae*), maintained on the model crop *Brassica oleracea*, to different types of cues from aphid lion (*Chrysoperla carnea*). The respective predation risks, we termed *Fear Factors*, were either lethal (consumption by predator), or non-lethal (non-consumptive predator-associated cues: plant-tethered predator cadavers and homogenised shoot-sprayed or soil-infused blends of predator remains). Our results show that the non-lethal risk cues differentially impeded prey reproductive success that varied by clone, suggesting genotype-specific response to fear of predation. Furthermore, whether plants were perceived as being safe or risky influenced prey responses as avoidance behaviour in prey depended on clone type. Our findings highlight that intra-specific genetic variation underlies prey responses to consumptive and non-consumptive effects of predation. This allows selection to act on anti-predator responses to fear of predation that may ramify and influence higher trophic levels in model agroecosystems.

## Introduction

Fear is an important drive for change in nature. It comes under different names, frequencies and intensities, and elicits a vast array of responses spanning the morphology, ontogeny, physiology and behaviour of ‘scared’ organisms^1–4^. The non-consumptive effects of predation in the context of the ecology of fear^5^ have been increasingly attracting attention^3, 5–7^. Exposure to predation threat has been reported to incur significant impacts on the fitness and phenotypes of animals across taxa^1, 4–6, 8^. For example, McCauley *et al*. (2011) remarkably showed the mere presence of a piscine predator, without access to prey, reduced dragonflies’ survivorship^9^. Moreover, predator-borne cues, which are traces, tracks and marks of a predator associated with its non-consumptive influence on prey^10, 11^, have been receiving increasing attention^8, 10–12^. For example, Ninkovic *et al*. (2013) demonstrated that bird cherry-oat aphid *Rhopalosiphum padi* avoided ladybird trails and excretions on plants. This advocated a potential for an insect-deterrent effect of predator’s signature (odour) that is expected to be dependent on predator numbers and signal strength^13^.

## Inducible reactions to uncertain circumstances

Animals face varied levels of uncertainty whilst they selectively examine the nature of risk in their environments, hence they react to non-lethal risk cues in comparison to fatal ones—as part of their varied adaptive responses to numerous selective pressures acting on them^14, 15^. This makes the assessment of risk by prey, in the presence of a threat nearby, complex and contingent on predator-associated cues, and thus will influence prey propensity to hide, fight or flight^2, 8, 12, 16, 17^. Although the expression of an anti-predator response to an imminent risk depends on the environmental context^18^ along with the type and the clarity of the perceived predation cues^19–21^, there are still gaps in our knowledge on the functionality of risk effects associated with predation^6, 22^. Furthermore, the perception and recognition of predator cues may induce plastic responses in prey striving to circumvent the risk of predation (*e.g*. ref. 23). However, in this regard, the role of prey’s within-species genetic variability in response to predation risk is still not well understood.

## Genetic variation in prey response to predator-associated cues

Even though non-consumptive effects of predation have been progressively reported (*e.g*. refs 3, 9, 11 and 24), little is known on the influence of intra-specific genetic variation in prey on the exhibition of altered response to predator-associated cues. It has been suggested that prey response to predation risk can be influenced by their intra-specific genetic variation^2, 25^, however, empirical data is thus far lacking. Moreover, only few studies endeavoured to spotlight the impact of non-lethal risk-associated cues on the performance of parthenogenetic model organisms of eco-evolutionary and economic significance (*e.g*. refs 13, 26 and 27) such as aphids^5, 27, 28^. Aphids reproduce exponentially under favourable conditions resulting in expanding colonies of congeners, sensitive and relatively adaptable to their changing environment^29–31^.

## Anti-predator responses entail ecological costs

In search for a safe and suitable plant, aphids probe the phloem of a potential host; a process that relies on the perception of gustatory cues that are vital for making the decision of whether to stay and establish a colony or relocate^32^. Phloem-feeders deselect less attractive or unpalatable plants to establish their population on the most suitable plant hosts^32, 33^. A presence of predation risk in the vicinity of aphid clones and the diffusion of alert signals throughout the population are shown to stimulate aphid mothers to express adaptive phenotypic plastic responses to threat (*e.g*. refs 31, 34 and 35). Disturbance inflicted via non-lethal cues can cause declines in aphid fitness, as it was demonstrated that aphid mothers may alter their reproductive success under stress^4, 26^. Moreover, aphids may demonstrate an adaptive control of morph production in response to the accumulation of dead conspecifics in the population under attack by a natural enemy^19^.

Once aphids settle on a suitable host and initiate the colonisation process, continuous phloem consumption is crucial for aphid survival^29^. Triggered population changes and time lags resulting from inducible predator-avoidance behaviours can lead to interruption of the constant feeding routine. This in turn can be costly for the aphids, particularly if the feeding gap and/or alteration of feeding routine are due to induced changes in fitness and behaviour owing to exposure to predation risk^4, 26, 27, 36^. A delay or malfunctioning in prey inducible response can also add to the costs^37^. In this regard, resulting changes in plant-aphid eco-evolutionary dynamics *per se* can ramify to affect ecosystem services relevant to crop productivity and cycles of elements^38–40^. An intra-specific genetic variation basis for anti-predator inducible defences as response to non-lethal predation risk has never been adequately studied before.

In this work, in order to examine the effect of within-species genetic variation of prey response to risk of predation, we respectively exposed four clones of green peach aphid *Myzus persicae* (Sulzer) to different types of lethal and non-lethal predator-related cues. On the 8^th^ day of the experiment, we investigated aphid reproductive success (final total number of aphids per enclosure), and aphid host preference for safe (non-treated) *vs*. risky (cue-treated) plants as a proxy for anti-predator avoidance behaviour. The predator-related cues were associated with the larvae of aphid lion *Chrysoperla carnea* (Stephens), and aphids were maintained on savoy cabbage *Brassica oleracea* L. convar. *capitata* var. *sabauda*. We use the term *Fear Factor* (FF) for five respective levels of predator-related risk treatments; see supplementary materials (Appendix 1, Fig. S1) for a conceptual graphical design: I) Risk-free level (FF0), the aphid alone treatment, where the predator and predator-associated cues were absent. II) Lethal *Fear Factor* (FF1), where a live predator foraged the enclosure. III) Non-lethal *Fear Factor* (FF2), where predator corpses were tied to the shoot-parts of one plant only of two available per enclosure (termed the “risky” plant), providing visual and olfactory stimulation. IV) Non-lethal *Fear Factor* (FF3), where a blend of predator remains was micro-sprayed on the shoot of one plant only (termed the “risky” plant) of two available per enclosure, providing olfactory stimulation. V) Non-lethal *Fear Factor* (FF4), where a blend of predator remains was micro-injected, at different depths, into the soil around the rootlets of one plant only (termed the “risky” plant) of two available per enclosure, providing olfactory stimulation. The *Fear Factor* treatments (FF2-FF4) comprised the predator-related, non-lethal risk effects under the focus of this work. See supplementary materials (Appendix 1, Fig. S1) for a conceptual graphical design.

We address the following questions:

1. Does the presence of predator-associated cues influence the reproductive success of prey in a clone-specific manner?
2. Do any clone-specific responses to risk stimuli vary across the applied different lethal and non-lethal predator-associated cues?
3. Do non-lethal predator-associated cues induce clone-specific avoidance behaviour in prey?

## Results

### Aphid reproductive success

**Figure 1:**
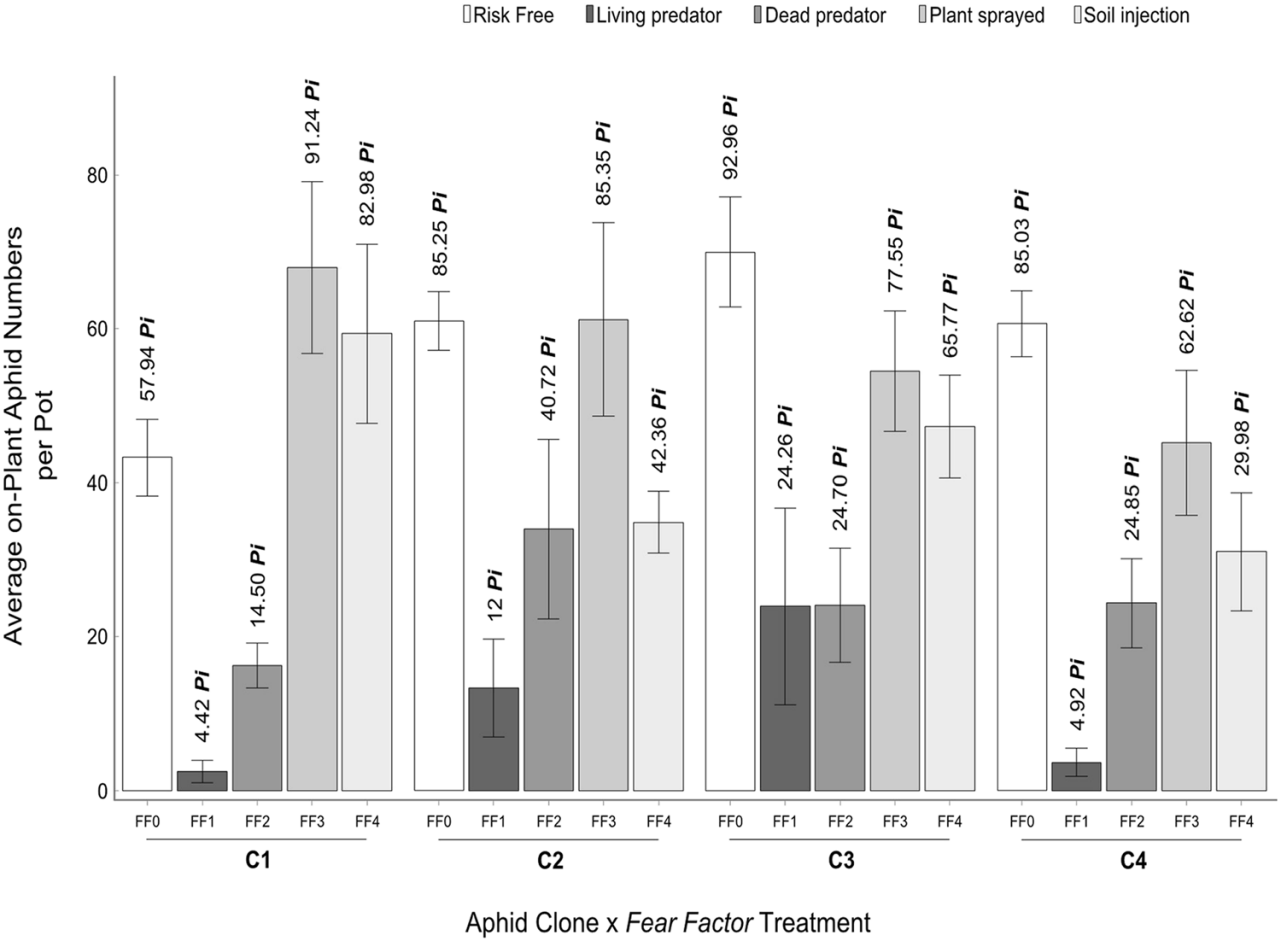
Aphid reproductive success. Average on-plant *Myzus persicae’s* total numbers per pot/enclosure (mean ±SE) are shown for the clones (C1–C4) across *Fear Factor* treatments. The bars are annotated with percentiles (*Pi*), which on the one hand relatively reflect the performance ranks (positions) of aphid reproductive success under different clone by *Fear Factor* combinations. On the other hand, the percentiles relatively denote the efficacy of the *Fear Factor* treatments in suppressing aphid reproductive success across aphid clones. *Fear Factor* levels are the absence of predation and predator cues (FF0 *i.e*. aphid alone, risk-free) and across the *Fear Factor* treatments [Living predator (FF1), dead tethered predator (FF2), shoot-sprayed cues (FF3), and soil-infused ones (FF4)].

There were significant effects of *Fear Factor* presence and treatments as well as plant risk status of being risky or safe in the enclosure on aphid reproductive success (measured as the total number of aphids counted per enclosure after 8 days from aphid introduction). Moreover, the interaction between aphid clone and each of these variables strongly influenced aphid reproductive success (Table 1, Fig. 1). Here, we annotated Fig. 1 with calculated percentiles (*Pi*) as a measure of the relative reproductive success (performance) of aphid clones across all possible encounters with the aphid lion or its bio-signature (see methods below). In general, FF1 (consumption by agile predator) and surprisingly FF2 (non-consumptive impact of visual-olfactory cues associated with predator corpses) treatments were effective in impeding aphid reproductive success in a clone-specific fashion. For example, we observed that the most reproductively successful clone under FF0 (the absence of predator and predator cues) was clone three (92.96 *Pi*), while the least successful one was clone one (57.94 *Pi*); a performance rank difference of 35.02 *Pi*, (Fig. 1). See supplementary materials (Appendix 1) for more information on the comparative performance, and supplementary materials (Appendix 1, Table S1) for further details on clonal differences in response to the different *Fear Factor* levels. Comparatively, under the influence of the non-lethal (non-consumptive) *Fear Factors* (FF3**-**FF4), all clones except, clone one, showed a general pattern of impeded reproductive success *i.e*. the clones differentially displayed reduced production of progeny in comparison to the FF0 reference frame. Moreover, the effect of FF3 varied across clones and was notable on clone four (22.41 *Pi* point drop in aphid performance rank, if compared with the reading under FF0), (Fig. 1). Conversely, FF3 was the least effective in suppressing aphid numbers in clone one, (Fig. 1); see also supplementary materials (Appendix 1). Moreover, treatment FF2 of the applied non-lethal predator cues was the first most efficacious in suppressing aphid reproductive success in a clone specific fashion, followed by FF4. On average, aphids of the four clones reproduced less when dead predators were present in FF2 and their responses varied considerably. The risk in FF2 (visual-olfactory in nature) had the most negative effect on clone one (4.42 *Pi*) followed by clone four (4.92 *Pi*), (Fig. 1). All in all, treatment FF4 (soil-infused cues) had a larger negative effect on aphid performance than FF3 (shoot-sprayed cues). Interestingly, aphids of clone one thrived under FF3 and FF4, contrary to the poorer performance of the other clones under these categories of risk, (Fig. 1).We also recorded numbers of aphids off-plant (signifying anti-predator dropping off plant behaviour), but found them to be negligible (nearly 0.7% of the whole population of aphids observed). The same applies for the production of alates (winged aphid morphs) that was also negligible (nearly 0.3% of the whole population of aphids observed).

**Table 1:** Aphid reproductive success in response to *Fear Factor*.

Explanatory Variables	Aphid reproductive success (final total numbers per enclosure)		
	χ^2^	DF	P
Aphid clone	6.837	3	0.077
*Fear Factor* absence/presence	61.615	1	**<0.0001**
*Fear Factor* Treatment	88.345	3	**<0.0001**
Plant Risk Status	301.530	1	**<0.0001**
Aphid clone × *Fear Factor* absence/presence	21.392	3	**<0.0001**
Aphid clone × *Fear Factor* Treatment	36.138	9	**<0.0001**
Aphid clone × Plant Risk Status	245.862	3	**<0.0001**

Analysis of *Myzus persicae’s reproductive* success as function of the absence/presence of *Fear Factor* (0, 1), (*Fear Factor*) treatments (FF1–FF4), and Plant Risk Status (safe or risky) across four aphid clones (C1–C4). Results are from a generalised linear mixed effects model (N = 200), with pot (enclosure) as a random factor. Significant values are shown in bold.

### Non-lethal *Fear Factor* effects on aphid host-plant preference

**Figure 2:**
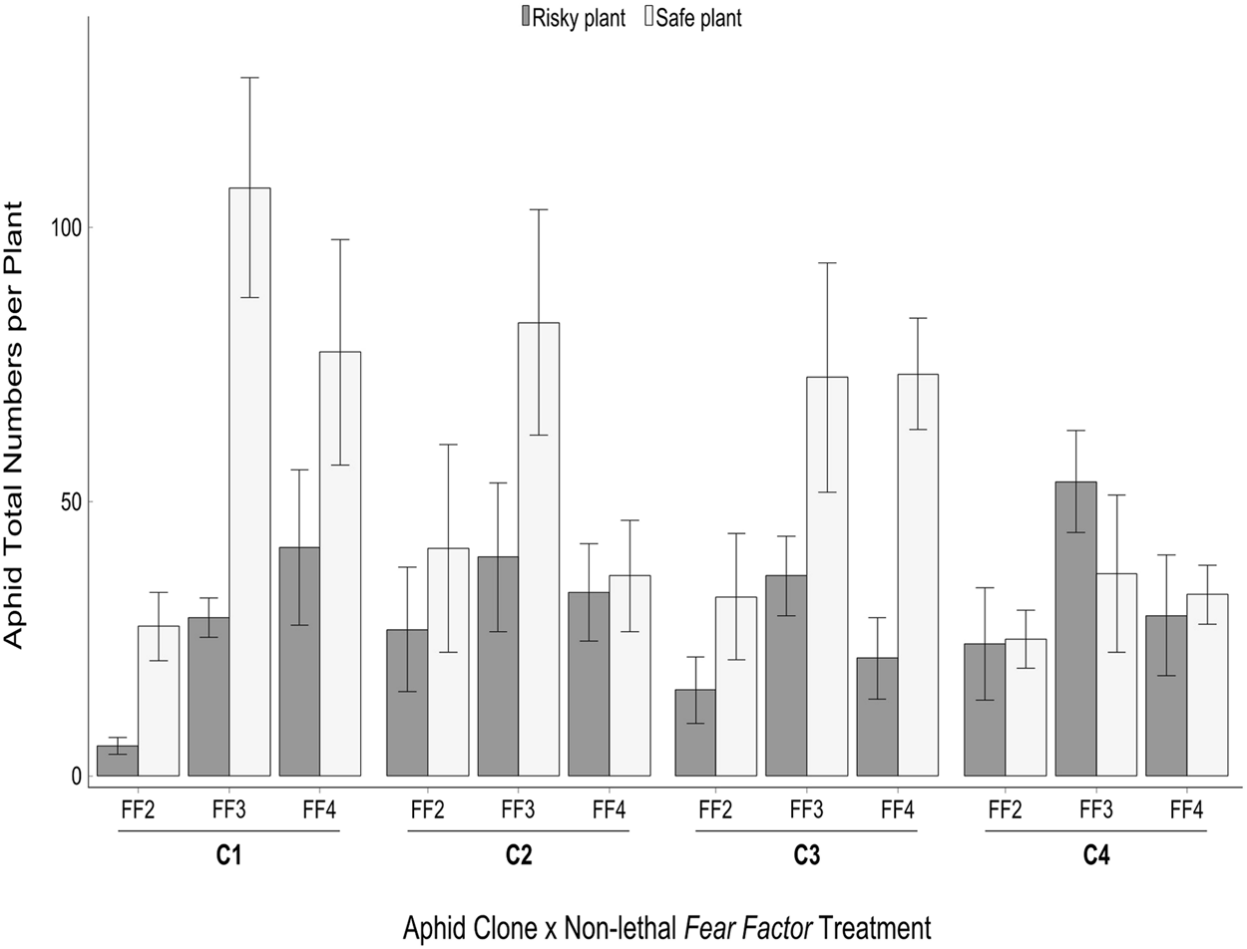
Aphid preference for safe *vs*. risky host plant after exposure to different levels of non-lethal predator-associated risk. *Myzus persicae* total numbers per plant (mean ±SE) across four clones (C1–C4) under different levels of non-lethal predator-associated cues (*Fear Factors:* FF2–FF4). Aphid clones were exposed to visual-olfactory cues associated with the dead plant-tethered predator (FF2), shoot-sprayed cues (FF3) and soil-infused ones (FF4). The safe plant (non-treated with *Fear Factor*) is depicted in blue while the risky plant is displayed in red.

Concerning aphid choice for safe *vs*. risky plants, only aphid clone significantly affected aphid preference (F = 5.994, DF = 3, P = 0.002); see supplementary materials (Appendix 2, Table S2) for a full display. In FF3, the general pattern for aphids was to favour the safer plant over the risky one, except one case, where clone four was on average noticeably more attracted to the cue-treated plants (Fig. 2). In the case of FF4, all clones distinguished between safe and risky plants, but the avoidance behaviour shown by clone two and four was less apparent than the one displayed by clones one and three. Under FF2, the choice to aggregate on the safer plant was evident for clones one, two and three, while clone four was the least discriminating one against the risky plants (Fig. 2).

We note that our results also show that in reaction to FF2 in few cases, aphids of clone two (two replicates), clone three (one replicate) and clone four (one replicate) aggregated on the farthest edge of few leaves of the risky plant. We also note that a second impulse of predator-associated cues in the middle of the infestation period [*i.e*. the second treatment application by shoot-spraying (FF3) and soil-infusion (FF4)] seemed to have induced a prolonged anti-predator response that manifested in persistent avoidance of the risky plants. Furthermore, during the course of exposure to the non-lethal lethal *Fear Factors*, the number of aphid cadavers was negligible, but exuviae were observed. See also supplementary materials (Appendix 3) for further details on the within-plant distribution of aphid clones in response to non-lethal *Fear Factors*.

## Discussion

In a predator-prey system, to the best of our knowledge this work is the first to use intra-specific genetic variability to examine prey ecology (reproductive success and behaviour) in response to non-lethal predator-associated risks. Using green peach aphid (*Myzus persicae*) as a model organism, we demonstrate that prey genotype can influence prey response to risk, with a notable decrease in reproductive success and pronounced risk-avoidance behaviour that varied among prey genotypes and across types of exposure to non-lethal predator cues.

Mobile predator larvae of the *Fear Factor* treatment (FF1) foraged enclosures in search for their prey. Our results show that (FF1) was the most effective type *Fear Factor* in suppressing prey numbers, in a clone-specific fashion, mainly directly through predation (Fig. 1). However, what is indeed interesting is that aphid reproductive success decreased under the influence of the tethered predator bodies (FF2), (Fig. 1), plausibly exerted via predator-related visual and olfactory cues^20, 27, 41, 42^. Also, the encounter with olfactory cues of the shoot spraying treatment and the soil-infusion treatment were differentially effective in reducing aphid performance. All in all, when contrasted with controls, shoot spraying with predator residues notably hampered aphid reproduction in almost 50% of the cases, while the cues of the plant-tethered predator cadavers (FF2) and soil-infusion (FF4) were universally largely effective in their negative impact across all the aphid clones of this work (Fig. 1). Our findings are in harmony with recent evidence for negative effects of predator trails and non-consumptive predator-associated cues on aphid fitness^3, 4, 9, 11, 26, 27^. Our results are also consistent with studies showing that visual and odour markings as predator cues can have dramatic effects on prey viability and population dynamics^41, 42^. When danger is perceived nearby, aphids emit alarm pheromones which in turn induce a variety of physiological, morphological and behavioural responses across aphid clones at risk^2, 27, 33^. The altered prey performance (reproductive success) in response to *Fear Factor* treatments, implying the effect of fear of predation^3, 10, 11^ was very notable (Table 1 and Fig. 1). In our study dropping-off plant (usually followed by desiccation, if relocation on a new host does not occur shortly after plant abandonment) did not seem to be a notable factor behind the reduction in aphid numbers, nor did overcrowding and within-clone competition, which were negligible during the eight days of the experiment. Furthermore, in our experiment the exposure to the non-lethal predator-associated cues was not prolonged up to the threshold, where aphid genotype would produce a significant number of alates (winged morphs)^2^. Hence, the decline in aphid numbers, subject to the presence of non-lethal predator cues, did not stem from investment in alates (dispersive but energetically costly and less fecund)^31, 36, 43^ or mortality due to food deprivation, but rather resulted from non-consumptive effects of predator-borne cues signifying fear of predation^3, 10, 11, 27^. This resulted in a differential decrease in aphid reproductive success due to perturbation, see also ref. 9.

The aphid lineages used in this work responded differentially to the applied predator-associated risk cues and the effect size varied by stressor (Fig. 1). Based on our results, we propose that part of the variation in prey performance can be attributed to differences stemming from intraspecific genetic variation and relevant phenotypic plasticity among conspecific genotypes^35, 44^, which occur in line with the behavioural responses further discussed below. The reduced numbers of aphids exposed to *Fear Factors* potentially indicated reduced fitness as a reaction to threat stimuli that were likely assessed, in a clone-specific fashion, by aphid mothers as signals of potentially dangerous environments for their forthcoming offspring^4, 26^. We adapt this logic to argue that the perceived risk induced particular changes in the performance of aphid mothers in response to disturbance/stress within uncertain and risky environments^4, 26^. Another factor that contributed to the a differential dwindled reproductive success across aphid clones was the costly anti-predator avoidance behaviour that consequently prevented aphids (in a clone specific fashion) from using the available resource in full and thus negatively affected their fitness (Fig. 2). Here, aphids on average differentially aggregated on the safe plant more than they did on the risky one per enclosure for the *Fear Factor* treatments (FF2–FF4). Based on the findings provided by Mondor *et al*. (2005) on offspring conditioning in cotton aphid (*Aphis gossypii*), a tangible decrease in aphid reproductive success can be partly attributed to the effect of non-lethal predation risk on aphid fitness and plasticity^35^. We assume a similar emergence of fear-steered decline in aphid populations to occur and risk-avoidance behaviour to be concurrently induced, where aphids are in contact with risk *i.e*. predator cues. However, the higher aphid numbers of clone one under non-lethal predator-related cues of olfactory nature (FF3 and FF4 treatments), and of clone two subject to FF3 (Fig. 1) can possibly be attributed to: i) the better ability of these clones to distinguish non-lethality of the predator-associated olfactory cues to which they were exposed, if compared with the aphids of the other clones (Fig. 1), ii) the ability of those clones to acclimatise faster to non-lethal risk (signified here by odour) than their conspecific clones, or iii) on the contrary, a general insensitivity to such type of olfactory cues. Note that the non-lethal *Fear Factor* treatments (FF2–FF4) were envisaged and deployed based on the increasing evidence on the impact of non-consumptive predator-borne cues on prey performance and behaviour^1, 10, 11, 13, 22, 45, 46^.

A fear-driven prey response may lead to modified calibration of plasticity in aphid daughters in response to threat^4, 26, 27, 31, 44^. These daughters are members of an aphid clone comprised by genetically identical copies of one genotype. As such, an alteration of aphid phenotype due to intrinsic and extrinsic factors may fall under the inclusive fitness theory. Here, less reproduction in a risky environment for the forthcoming offspring can be of fitness benefits for clone survival^44^. However, the induction and development of phenotypes, which may vary in fitness and in response to environmental risk can be apparent, as in our case, and are expected to occur across taxa (see ref. 5).

Our findings show that the presence of predation risk may drive aphids to agglomerate on the safer plant; and such response was clone-specific (Fig. 2). Here, under the non-lethal *Fear Factor* treatments, aphids had the freedom of choice to select between two options available per pot/enclosure; a “safer” or a “riskier” plant. Almost ubiquitously, the vast majority of the populations of each clone favoured the safer plant, following an exploration of the enclosed environment (Fig. 2). Staying off riskier plants or sticking to refugia (relatively safer spots in enclosures *versus* risky ones) is usually an effective anti-natural enemy strategy (*e.g*. ref. 47).

The perception of risk in the vicinity of an aphid clone will influence aphid preference and resource utilisation^8^. In the case of the non-lethal threat (FF2), the dead predator, tied to the plant stems, was technically a mass of gradually decaying matter through the duration of the investigation, but bore the visual and olfactory signature of an aphid’s natural enemy. The deterrence effect in this case was enough to keep the majority of mother aphids and their coming progeny at a distance during the relatively short period of exposure; an observation which receives support from the risk allocation hypothesis *sensu* Lima and Bednekoff^48^. However, the specific gregariousness observed, *e.g*. in clone two and three, where fewer aphids densely aggregated on the edge of risk on the risky plant under the FF2 treatment, indicated either tolerance/habituation for the non-fatal predation risk or a degree of risk-taking. Here, aphids aggregated on the safest portion of a potentially highly risky plant, despite having the choice to share enough room with their risk-avoiding kin on the safer plant in the enclosure. The colonisation at the verge of potential risk indicated a transition in aphid risk assessment, where colonisers showed more risk-taking propensity. In the rise of evidence for aphid personality variation (*e.g*. ref. 49), such peculiar propensities can be conceived as a variant of adaptive behavioural plasticity and could be attributed to aphid identity^49^ and the social aggregation amongst aphids on those venues^50^, see also ref. 39.

The observation of the overall aphid aggregation prior to the application of the second pulse (in FF3 and FF4) revealed that the vast majority of aphids, except the congeners of clone four, were avoiding the risk and thus risk-averse (Fig. 2). Relative to its counterparts, aphid clone four showed the least propensity to discriminate against the risky plants, indicating the least avoidance behaviour (particularly under the effects of the cues of olfactory nature of FF3). This can potentially be attributed to risk-taking or habituation as aphids of this clone made their decision to gradually inhabit the risky plant, regardless of the potential predation risk. From a different perspective, aphids of clone four, under the influence of the cues of olfactory nature in FF3, were more able to tolerate the non-lethal predation threat distinguished by olfaction. However, despite being able to tolerate or habituate to non-lethal predation threat, the clone four’s risk-taking behaviour (Fig. 2) led to notable decrease in its reproductive success across the non-lethal *Fear Factor* treatments (compared to its clonal counterparts). Note that in the absence of risk, clone four ranked third in performance (Fig. 1). Such induced aphid response to threat stimuli can be communicated amongst members of the clone both horizontally and vertically via maternal preconditioning of offspring^51, 52^ following hereditary and non-hereditary routes^31, 52, 53^. Inter-generational communication may be a strategy of insect prey to avoid risky plants; with evidence for the ability of sap-feeding insects to learn to avoid environmental risk^33^.

What is counter-intuitive, however, is that clone one performed better under FF3 and FF4 (than under FF0), whilst showing remarkable avoidance behaviour of the risky plants under those categories of *Fear Factor*. This indicates that such anti-predator behaviour might have provided the means whereby aphids of clone one thrived under non-lethal predation risk.

It can be argued that the persisting discrimination of the risky spots across the *Fear Factor* treatments, comprising the non-lethal risk, indicates a choice made by aphids through *a priori* assessment of the potential risk cues hence allocating and thus avoiding risk from the beginning of the settlement process^48^. As such, the persistent pattern of warding off potential threat indicates an enemy-evasion tactics *i.e*. inducible risk avoidance response by keeping away from the scent and/or visual aspects of threat in our examples. Nevertheless, our *M. persicae* clones had no contact with any predation threat for several months before the experiment took place. As such, being naïve prey, expressing anti-predator behaviours (avoidance) within a venue carrying novel predator-borne cues^54, 55^ could be described as an agnostic behavioural response that bears innate submissive nature (*i.e*. retreat) against aphid lion^55, 56^. The lethal and non-lethal cues stimulating agonism, in terms of conflict avoidance, against natural enemies as an inducible prey defence are not yet well understood.

The induced response of the aphids exposed to risk can also be attributed to the effects of predator signature such as semiochemicals which are attracting, repelling or deterring chemical signals emitted by an organism (releaser) and are known to modify specific traits of the recipient organism^54^. Upon being perceived by insect prey, these signals can interfere with the insect communication, alter their behaviour and reduce their fitness^54^.

Still, the non-lethal predation risk in the case of the tethered predator, with associated visual-olfactory cues, in our work, was the most effective in suppressing aphid numbers for all clones; in comparison with the other types of non-lethal *Fear Factors* bearing only olfactory cues (the shoot-spraying and soil-infusion), (Fig. 1). This indicates that aphids can be more sensitive to the combination of visual and olfactory cues associated with non-lethal predation risk because this likely makes the composite stimulus less ambiguous and hence gives a stronger signal of the imminent risk that is otherwise unpredictable^20^.

In this work, the *Fear Factor* not only influenced aphid performance (reproductive success), but also aphid choice to populate the riskier *vs*. safer plants [supplementary materials (Appendix 2, Table S2)]. This represents a trade-off between cost and benefit of stay or escape, analogous to the findings reported by Weisser *et al*. (1999)^2^ and Siepielski *et al*. (2016)^57^, where disruption of feeding can be costly for the highly host-dependent prey such as aphids, with consequences on their fitness and the very environments they live in and interact with^4, 26, 27^. As such, fear of predation can alter prey population dynamics, demographics and spatial occupation of available resources with far-reaching impact on ecosystem structure^58^. The influence of the non-lethal *Fear Factors* can amplify through trophic systems and may affect agro-ecosystem functions and services; with socio-economic dimensions^38, 59^. In sum, our findings are in accord with the view that (fear of predation) can have a magnitude similar to predation effects and thus should not be ignored^3, 5, 6, 60^.

## Conclusion

As far as we know, this is one of the first studies to provide solid evidence for within-species variation underlying prey response to predator-associated cues. The novel result of different responses among genotypes to risk may have general implications, as different species of other prey taxa may also likely have genotype-specific responses to fear of predation. Within-species genetic variation effect may be as great as the variation in responses among species. This necessitates future investigation of the differential suppression of prey reproduction and the emergence of antipredator defences in different model agroecosystems to further our understanding of the nature of such a phenomenon. In the light of intra-specific genetic variation in parthenogenetic prey naïve to predation, we also demonstrate that non-lethal risks have strong effects on deterring aphids and decreasing their performance. The vast majority of aphids in this study developed a distinct behavioural clone-specific responses to encounters with trails and remainders of their predator, although these encounters and the cues associated with them were non-lethal *i.e*. non-consumptive. We advocate the inclusion of fear of predation as a variable in the investigation of the eco-evolutionary dynamics of predator-prey systems in future surveys. Our findings add to the increasing evidence on the ecology of fear and non-consumptive effects of predation risk and have theoretical and experimental utility in the study and regulation of crop pests. The application of predator residua (remains, soil-infusion or spray) may provide environmentally as well as human-consumer friendly insecticide alternatives that deserve further testing in larger eco-agricultural settings.

## Methods

### Aphid clones and host plant

*M. persicae* is a serious plant-virus vector and a polyphagous pest of a wide range of cash crops^28, 29, 61^. Being a phloem-feeding insect, *M. persicae* is highly dependent on ﻿the host plants, where aphids reside, feed and reproduce^29^. Under temperate conditions, *M. persicae* is viviparous (*i.e*. producing living young not eggs) and it replicates itself through parthenogenesis creating a population of genetically identical females referred to as a clone (anholocyclic life cycle, no sexual reproduction). In the wild, eggs are only produced for the purpose of overwintering under harsh conditions. Low temperatures may trigger *M. persicae* to produce mating morphs to complete the life cycle (holocyclic) that only takes place on winter-host trees (*Prunus spp*.)^29, 62^. *M. persicae* is highly polymorphic in colour, producing green and pink morphs^63^. It has been demonstrated that aphids rely on more than one type of perception to assess their environment and thus they show multimodality of perception^20^ and can adjust their responses and preconditioning of their offspring dependent on the clarity and frequency of the information they obtain form their environment^20, 29^.

We used four different clones of *M. persicae*. One green clone was reared starting from one individual of a lineage sample obtained from the JKI (Julius Kühn-Institut, Berlin, Germany). Whereas, the other three aphid clones were each raised from a single individual respectively of samples provided by the Bayer CropScience AG (Monheim, Germany). The pink clone (C1) was collected on cultivated tobacco (Japan), the green clone (C3) isolated from pepper plant (Monheim, Germany), and green clone (C4) is a screening strain of the Bayer Company (Bonn, Germany).

Aphids were maintained on Chinese cabbage *Brassica rapa* ssp *chinensis* L. for several generations before being used to infest enclosed potted savoy cabbage *B. oleracea*. Plant seeds were purchased from a commercial supplier in Germany and two seeds were sown at opposing sides of each pot. Plants were ready for infestation at three weeks after germination under mesic conditions (18-hour daylight, ~24°C). Each of the 6″ pots was enclosed with a 16″ transparent acrylic cylindrical sleeve with fine-meshed windows for ventilation. Thirty 4^th^ instar nymphs of each aphid clone were added onto the soil surface in the central part of the enclosure in order to give the instars the freedom to choose between the two available host plants. Aphids were left for one hour therein to settle on their plant of choice before exposure to the particular risk in question. Aphids were placed with a fine damp brush on the soil in the middle of each pot/enclosure and left to make their choice for colonising a host plant between each of the two plants available.

### *Fear Factor* treatments

We define the *Fear Factor* treatments, (**FF**) as the exposure to the lethal (consumptive) and non-lethal (non-consumptive) risks associated with aphid lion. This was either an exposure to lethal (live predator) or nonlethal cues (predator’s signature). Aphid lion larvae were procured from a local supplier (Katz Biotech AG), Germany. These predacious larvae make a potent aphidophagous agent in green house settings^64^. Aphids used in this work were naïve (inexperienced with predation risks). They were raised and maintained for several generations in moderate densities and under preferable conditions in the complete absence of any encounter with natural enemies. The following levels of *Fear Factor* (**FFn)** were applied individually for each of the four aphid clones used:

**FF0: Risk-Free (Aphid only, absence of predation and predation risk)**. Enclosures with each of the four aphid clones maintained alone *i.e*. risk-free under no predation and predator-related cues.

**FF1: Lethal mobile (actively foraging) predator**. One aphid lion larva was added per enclosure infested with thirty aphid 4^th^ instars shortly introduced beforehand.

**FF2: Non-lethal plant-bound dead predators**. Aphid lion larvae were euthanised by freezing before being tethered individually with a thread to one stratum (top third, mid third and bottom third) of one of the two plants available per pot (randomly selected). We note that our aim here was to actually use general predator-borne cues (marks/residua), with different allocation of non-consumptive environmental risk for aphids. As such, we did not aim for mimicking a certain predator type (sit-an-wait *vs*. actively hunting). The non-lethal predator-associated cues in this treatment were estimated to be of both visual and olfactory nature, with evidence that aphids rely on multi-modal perception of their surroundings^20^, see also ref. 65.

**FF3: Non-lethal, shoot-sprayed predator-associated cues**. A predator body solution (0.35 larvae of *C. carnea* / ml distilled water) was prepared by homogenising frozen larvae in distilled water. The solution was micro-sprayed on one plant only (randomly selected) of the two plants available per pot, while the other plant was protected by an acrylic sheet as a separator. We sprayed 6 ml of the solution as evenly as possible on the plant shoot. The acrylic separator was removed 5 minutes later and we introduced thirty aphids (4^th^ instars) to each enclosure as above. We repeated the spraying procedure after four days using a freshly prepared solution as above. Blending the euthanised bodies of the predator into an aqueous solution provides the empirical advantage of utilising the bio-signature of the predator as a spreadable non-lethal risk cue (*e.g*. ref. 3). The non-lethal risk-associated cues in this treatment were estimated to be invisible and of olfactory nature.

**FF4: Non-lethal, soil-infused predator-associated cues**. The same solution of the FF3 treatment was applied anew via several micro-infusions with a modified pipette adjacent to the rootlets, at varying depths and on the soil surface where the stem emerges; and applied to one plant only per enclosure (randomly selected). This was followed by the introduction of aphids as described in FF3. We repeated the infusion procedure after four days using a freshly prepared solution. We predicted an aphid-deterrent buffer zone of chemical cues (likely olfactive) to encircle the base of the emerging stem that is expected to create a perceivable non-consumptive predator-related stimulation^1, 3, 65, 66^. The stimuli of this treatment were non-visual and estimated to be of olfactory nature. We also speculated that an indirect influence of the predator solution could be plausibly mediated through host plant.

### Plant risk status

All the plant pairs per pot/enclosure in our study were always adequately apart from each other and the same distance between the two plants in a pot was fixed in each and every replicate of each FF treatment [see supplementary materials (Appendix 1, Fig. S1) for a conceptual graphical design]. Moreover, we placed the enclosures on different tables with enough distance (16″) between enclosures to eliminate any adjacency. All plants within the risk-free category (FF0) were considered safe due to the absence of *Fear Factors*. Whereas, all plants within the FF1 category of the risk treatment were considered risky due to the agility/mobility of the aphid lion, which actively roamed the resource it was enclosed in and hence left predator-borne cues on both plants in the enclosure. Under the non-lethal risk treatments (FF2, FF3, and FF4) the whole enclosure was potentially risky with more concentration of the predator cues on one plant of two available per enclosure. Thus aphids were to choose between a plant treated with the *Fear Factor* and therefore estimated to be a *risky* choice and a non-treated, relatively safer, one within the enclosure. As such, we defined a plant status for being risky or safe. We used this factor to enhance the precision of the analysis of aphid reproductive success (Model 1 below). See supplementary materials (Appendix 1, Fig. S1) for an illustration of the conceptual design of the experiment.

The experiment lasted for eight days after commencing the *Fear Factor* treatments. Aphid introduction was synchronised, with the same specific number and the same age per enclosure as described above. On the day of data collection, we measured the following parameters: Aphid reproductive success (measured as the total number of aphids counted per enclosure after 8 days from aphid introduction) and host-plant preference (aphid numbers on each plant, signifying aphid choice for aggregation on risky *vs*. safe plants per enclosure *i.e*. anti-predator avoidance behaviour). We also further examined aphid distribution within host plant, and we provide supportive insights on this complementary investigation via supplementary materials (Appendix 3, Table S3 and Fig. S3). The length of the experiment was carefully selected to reflect the fact that fresh predator-related cues are more reliable for aphids to make decisions and adjust anti-predator responses than older cues (see ref. 17). The older the cues (bio-signature of the predator), the less accurate information they provide and thus may become insufficient, by time, to induce alteration in aphid response^20^. Since the average generation time is approximately14 days and, in general, the average life span is 23 days and may last longer^20, 29, 62, 67^, the duration of 8 days of the experiment also ruled out an overlap of successive generations and thus over-crowding was prevented.

### Statistical analysis

We analysed the *Fear Factor* effects on aphid reproductive success and aphid host-plant preference (anti-predator avoidance behaviour) across four aphid clones using R 3.2.0^68^. We performed the analysis using the two following models:

#### Model 1: Effect of Fear Factor presence, Fear Factor treatment and Plant risk status on aphid reproductive success

We applied a generalised linear mixed effects model (Poisson family to account for non-normality in residuals). Here pot (enclosure) was a random factor, and we applied the *glmer* function (with ‘bobyqa’ optimisation algorithm) using the R packages *lme4* ^69^ and *car* ^70^. We used 200 plants (5 **FF** treatments x 4 aphid genotypes x 5 replicates x 2 plants per replicate).

We tested the effects of aphid clone (to quantify the effect of aphid intraspecific genetic variation), absence/presence of *Fear Factor* [FF (0,1)], *Fear Factor* treatments (FF1–FF4), plant risk status (risky or safe) and the interactions of these effects with aphid clone on aphid reproductive success. Furthermore, we calculated the z-scores matching the performance of each clonal line across all treatments using the equation 1:

1 $$z\mbox{-}{\rm{s}}{\rm{c}}{\rm{o}}{\rm{r}}{\rm{e}}=({\rm{X}}-\mu )/\sigma ,$$

where X is an observed value, **μ** and σ are mean and standard deviation respectively.

This was followed by converting the z-scores into percentiles (*Pi)* in order to comparatively quantify the performance rank (percentiles) of the focal clones across *Fear Factor* treatments, and to illustrate the relative efficacy of the applied **FF** treatment in suppressing aphid performance (reproductive success). See supplementary materials (Appendix 1) for supportive information on aphid comparative performance, and supplementary materials (Appendix 1, Table S1) for posthoc Tukey HSD test providing further details on clonal differences in response to the different *Fear Factor* levels.

#### Model 2: Non-lethal risk effects on host-plant preference (Anti-predator avoidance behaviour)

We analysed aphid host-plant preference per enclosure under the effects of the non-lethal *Fear Factors*, across aphid clones. Here we used a generalised linear model with a quasibinomial family (*glm* function of the *lme4* package). FF0 (risk free *i.e*. absence of *Fear Factor*) and FF1 (lethal predator) were excluded from the dataset for this analysis. Here we investigated the differential effect of the non-lethal *Fear Factor* types pertaining to the state of being visual-olfactory (treatment FF2) or invisible and of olfactory nature (treatments FF3 and FF4, separate). Here aphids made a choice to populate a plant treated with predator-borne cues or another one in the enclosure that was non-treated with the *Fear Factor*. We used 120 plants (3 **FF** treatments x 4 aphid genotypes x 5 replicates x 2 plants per replicate).

The datasets generated during and/or analysed during the current study are available in the [Figshare] repository, [https://figshare.com/s/347911ba998dfb96d24e].

## Electronic supplementary material

Supplementary Information
