# Supplementary material for: Fear of predation alters clone-specific performance in phloem-feeding prey
Source: Sci Rep. 2017 Aug 9;7:7695. doi: 10.1038/s41598-017-07723-6 (PMC5550486; doi:10.1038/s41598-017-07723-6)
Supplement: Supplementary file 1 — Supplementary Information [file 41598_2017_7723_MOESM1_ESM.pdf]

Online Supporting Information

Supplementary materials, Appendices 1, 2 and 3

Fear of predation alters clone-specific performance in phloem-feeding prey

Mouhammad Shadi Khudr, Oksana Y. Buzhdygan, Jana S. Petermann, Susanne Wurst

Supplementary materials, Appendix 1

Appendix 1. Percentiles utility

We standardised the average aphid total numbers per enclosure of each aphid clone across all clone by risk (*Fear Factor*) treatment combinations, by converting the means of each aphid clonal reproductive success (performance) into z-scores based on the equation:

$$\text{z-score} = (X - \mu) / \sigma$$

, where X is an observed value, $\boldsymbol{\mu}$ and σ are mean and standard deviation respectively.

This was followed by converting the z-scores into percentiles *Pi*. The calculated percentiles provided comprehensive supportive information on the ranking positions (performance rank) of each aphid clone across varying levels of the applied predator-associated risk (*Fear Factor).* The use of percentiles *per se* provided comprehensive comparison of aphid performance and *Fear Factor*s efficacy as percentiles enable better communication of data since this approach allows for relative understanding of the standing of values/groups (clones, here) *i.e.* their performance rank across all possible encounters with the aphid lion or its cues (bio-signature)[1,2,3]. Percentile ranks were integrated in the visualisation of our data in Fig.1.

Detailed explanation of Fig. 1 (article main text) in the light of performance ranking

There is approximately 20 percentile (*Pi*) point-range difference between clone one x FF1 and clone three x FF1 (where aphids were the fittest under this category of the *Fear Factor*). It is notable that aphid performance relevant to the exposure to the olfactory risk cue of FF3 was higher in rank than the performance under the risk free (FF0) for clone three, while performance rank of clone two remained roughly unchanged under FF0 and FF3 (85 *Pi*). Under FF2 category of the *Fear Factor* the magnitude of the visual-olfactory risk effect on clone three was quite similar to its magnitude on clone four and the performances of both aphid clones under this risk category were nearly identical with the rank of clone three under FF1. Yet, the

biggest difference between FF1 and FF2 was in clone two (performance rank shift of 30 *Pi*). It is noteworthy that the performance rank of that clone subject to FF4 was next to its rank under FF2 (with 2.4 *Pi* difference). Moreover, clone one's performance rank under FF3 was the closest to clone three's rank under FF0 (Risk free). Similarly, clone two under FF3 and clone four under FF0 had almost identical performance ranks. See main text results and Fig. 1.

References

1. Costanza, M., Galobardes, B. & Morabia A. Using percentiles to summarise data instead of means and standard deviations. *Soz. Praventivmed.* **47**(6): 427-429 (2002).

2. Nour, A. Quantifying the impact of transit reliability on users cost - A simulation based approach - Master's thesis. (The University of Waterloo, Canada, 2009). Retrieved from https://uwspace.uwaterloo.ca/bitstream/handle/10012/4616/Nour_Akram.pdf?sequence=

3. Estabrook, G. *A computational approach to statistical arguments in ecology and evolution.* (Cambridge University Press, Cambridge, 2011).

Appendix 1. Fig. S1. Conceptual diagram of the experimental design is available in the [Figshare] repository, [https://figshare.com/s/c2d3981a2d3830d411a2].

Appendix 1. Fig. S1. Conceptual diagram of the experimental design. We used four different aphid clones of *Myzus persicae* (thirty aphid 4th instars of each clone per enclosure) on potted savoy cabbage *B. oleracea* (two plants per enclosure). The two plants were seedlings (3-week old after germination) and were systematically sown apart at opposing ends of the pot across treatments. Aphids were always dropped onto the soil in the middle space between the two seedlings using a damp fine brush. The following *Fear Factor* treatments were applied: **Risk-Free (FF0)** means aphids were alone without predation threat (predator and predator-related cues were absent). **Lethal (actively foraging) predator (FF1)** means one aphid lion larva was added per enclosure infested with thirty aphids (4th instars) shortly introduced beforehand. **Non-lethal, plant-bound dead predators (FF2)** means that aphid lion larvae were euthanised by freezing before being tethered individually with a thread to one stratum (top third, mid third and bottom third of the shoot) of one plant only (randomly selected) of the two plants available per pot/enclosure. **Non-lethal, shoot-sprayed predator-associated cues (FF3)** means a predator body solution (0.35 larvae of *C. carnea* / ml distilled water) was micro-sprayed on one plant only (randomly selected) of the two plants available per pot/enclosure, while the other plant was protected by an acrylic sheet as a separator during the micro-spraying. **Non-lethal, soil-infused predator-associated cues (FF4)** means the same solution of the FF3 treatment was applied anew via several micro-infusions adjacent to the rootlets, at varying depths and on the soil surface where the stem emerges; and applied to one plant only per enclosure (randomly selected). Under the non-lethal risk treatments (FF2, FF3, and FF4) the whole enclosure was potentially risky with more concentration of the predator cues on one plant (risky) in comparison to the other non-treated plant (safe). All plants within the risk-free category (FF0) were considered safe due

to the absence of *Fear Factors*, whereas, all plants within the FF1 category were considered risky due to the agility/activity of the aphid lion.

Appendix 1, Table S1. Posthoc Tukey HSD test for the (aphid clone by Fear Factor treatment) multiple comparisons of the clonal reproductive success is available in the [Figshare] repository, [https://figshare.com/s/ef34a4d54fa30b720b46 **].**

Appendix 1, Table S1. Posthoc Tukey HSD test for the (aphid clone by *Fear Factor* treatment) multiple comparisons of the clonal reproductive success. Following Model 1 (described in the main text methods, see also results and Table 1 therein), we applied a Tukey HSD test, using R 3.2.0[1], 'multcomp' package[2], to show all the pairwise comparisons for each *Myzus persicae's* clone and across clones (C1-C4) in terms of their reproductive success in response to *Fear Factor* treatments (FF0-FF4).

References

1. R Development Core Team. *R: A language and environment for statistical computing*. R Foundation for Statistical Computing. (Vienna, Austria. ISBN 3-900051-07-0, 2013). Retrieved from http://www.R-project.org

2. Hothorn, T., Bretz, F., & Westfall, P. Simultaneous inference in general parametric models. *Biometrical j.* ***50***(3), 346-363 (2008). http://dx.doi.org/10.1002/bimj.200810425

Supplementary materials, Appendix 2

Appendix 2, Table S2. Aphid preference for host plant per enclosure (safe/risky) in response to non-lethal predator cues. Analysis of *Myzus persicae's* avoidance behaviour (preference to aggregate per plant per enclosure) as function of the non-lethal predator-associated cues of the *Fear Factor* treatments (FF2 − FF4). Visual-olfactory cues in FF2, olfactory cues in FF3 and non-visual (mainly olfactory) cues in FF4. Results are from a generalised linear model with quasibinomial family (N=120 plants resulting from 3 **FF** treatments x 4 aphid genotypes x 5 replicates x 2 plants per replicate). Plant were contrasted per pot/enclosure (*Fear-Factor* treated or non-treated). Significant values are shown in bold. See main text methods for more details. Also, see Appendix 1 (Fig. S1) for a conceptual graphical design of the experiment.

Explanatory Variables	Aphid Preference (safe plant or risky plant per enclosure)			
	SS	DF	FF	P
Aphid clone	257.71	3	5.994	**0.002**
Non-lethal *Fear Factors*	7.51	2	0.262	0.771
Aphid clone x Non-lethal *Fear Factors*	75.68	6	0.880	0.517

Supplementary materials, Appendix 3

Insight on the detailed aphid within-plant distribution

As explained in the text body of the article, the experiment lasted for eight days after commencing the predation risk treatment (*Fear Factors*). We aimed at deciphering the behaviour of *Myzus persicae* further by investigating within host-plant distribution that is aphid choice to aggregate on plant-shoot parts: *i*) stem, *ii*) above leaves, and *iii*) below leaves. Herein, we address the following question:

Subject to different non-lethal predator-associated cues, does within-plant distribution vary on a clone-specific basis?

The explanatory variables were: 1) Aphid intraspecific genetic variation (clone effect comprised by 4 aphid conspecific lineages). 2) Non-lethal *Fear Factor* (FF) composed of visual-olfactory predator-associated cues (FF2), and olfactory predator-associated cues of FF3 and FF4, respectively, (see also the main text methods). 3) Plant risk status for being safe (non-treated with FF) or risky (FF-treated).

Using R 3.2.0[1], we investigated aphid behaviour (within-plant distribution on shoot parts) under the aforementioned effects, where we applied a multinomial log-linear model (*multinom* function of the '*nnet'* package[2]; where the likelihood is conditional) supported by the R package 'car'[3]. Aphid within-plant distribution was highly influenced by aphid intraspecific genetic variation, non-lethal risks and their interaction (Appendix 3, Table S3). The effect of plant risk status on aphid behaviour was not significant. However, the effects of the interaction between aphid clone and plant risk status on aphid affinity for certain shoot parts was marginally insignificant (Appendix 3, Table S1). The non-lethal risk treatments led to similar within-plant distribution patterns *i.e.* in general, aphids confined themselves less to stems (8± 1.4% SE) and leaf upper-sides (27± 1.9% SE), while the majority resided below

leaves (64 ± 2.0% SE). However, aphid affinity for certain shoot parts varied concordant with plant risk status and aphid identity. For example, in FF2, proportions of aphids on the stem of the safe plant *vs.* the risky one were different for all clones but clone four (Fig. S3). Aggregation above-leaf, under FF3, varied between the safe plant *vs.* the risky one and across clones, but aphids of clone four showed consistent aggregation on the upper-sides of leaves on both plants per pot/enclosure. As such, the non-lethal *Fear Factors* not only influenced aphid performance and preference (see the main text results), but also modulated within-plant distribution (above-below leaves or stems), across aphid clones.

Appendix 3, Table S3. Aphid within-plant distribution (on shoot-parts) subject to non-lethal *Fear Factor* (FF) treatments, across four clones of *M. persicae* (C1-C4). Non-lethal risks = *Fear Factors* (FF2, FF3 and FF4). Results are from a multinomial log-linear model (N=360 [three shoot parts: *i*) stem, *ii*) above leaves, and *iii*) below leaves] of 120 plants [3 FF treatments x 4 aphid genotypes x 5 replicates x 2 plants per replicate]). Significant values are shown in bold.

Variables	Within-plant distribution of aphids		
	LR_{χ}^{2}	DF	P
Aphid clone	95.402	6	**< 0.0001**
Non-lethal *Fear Factors*	30.274	4	**< 0.0001**
Plant Risk Status	3.087	2	0.214
Aphid clone × Non-lethal *Fear Factors*	86.365	12	**0.0001**
Aphid clone × Plant Risk Status	11.614	6	0.071

Appendix 3, Fig. S3. Within-plant distribution of aphid clones subject to non-lethal risks (Fear Factors [FF2, FF3 or FF4]) is available in the [Figshare] repository,

[https://figshare.com/s/a83d3223953609020b3d].

Appendix 3, Fig. S3. Within-plant distribution of aphid clones subject to non-lethal risks (*Fear Factors* [FF2, FF3 or FF4]). Aphid average percent on stem, above-leaf and below-leaf on safe (F) *vs.* risky (*R*) plants per non-lethal risk treatment is illustrated across four clones of *M. persicae* (C1-C4). Each pot/enclosure included a non-treated plant *i.e.* safe (S) and a treated one with a respective *Fear Factor i.e.* risky plant. Aphid clones were exposed to the following cues of non-lethal risks (*Fear Factors*)*:* visual-olfactory cues associated with the plant-tethered dead predator (FF2), and cues of invisible nature (olfactory): shoot-sprayed cues (FF3) and soil-infused ones (FF4).

References

1. R Development Core Team. *R: A language and environment for statistical computing*. R Foundation for Statistical Computing. (Vienna, Austria. ISBN 3-900051-07-0, 2013). Retrieved from http://www.R-project.org

2. Venables, W. & Ripley, B. *Modern applied statistics with S.* Fourth Edition. (Springer – New York. ISBN 0-387-95457-0, 2002)

3. Fox, J. & Weisberg, S. *An {R} Companion to applied regression* (2nd ed.). (Thousand Oaks CA: Sage, 2011). Retrieved from http://socserv.socsci.mcmaster.ca/jfox/Books/Companion
